# Reliability of the Harris Hip limping sub-score in patients undergoing total hip arthroplasty

**DOI:** 10.1007/s00264-023-06082-4

**Published:** 2024-01-13

**Authors:** Kevin Rose-Dulcina, Xavier Gasparutto, Az-Eddine Djebara, Morgan Gauthier, Matthieu Zingg, Anne Lübbeke, Stéphane Armand, Didier Hannouche

**Affiliations:** 1 Kinesiology Laboratory, Geneva University Hospitals and University of Geneva, Geneva, Switzerland; 2 Division of Orthopaedics and Trauma Surgery, Geneva University Hospitals and University of Geneva, Geneva, Switzerland; 3 Nuffield Department of Orthopaedics, Rheumatology and Musculoskeletal Sciences, University of Oxford, Oxford, UK

**Keywords:** Reliability, Clinical score, Limping assessment, Gait, Total hip arthroplasty

## Abstract

**Purpose:**

In patients undergoing total hip arthroplasty, limping is a significant symptom, often assessed with the limping sub-score of the Harris Hip Score. However, the reliability of this sub-score has not been specifically investigated. The purpose of this study is to investigate the intra- and inter-rater reliability of this sub-score.

**Methods:**

Thirty patients undergoing THA were recruited and performed a gait analysis before surgery and three months after surgery. In addition, 30 asymptomatic participants were included. In total, 90 visits were analysed in this study. The HHS limping sub-score was assessed for each visit using a video (front and back view side-by-side) of a ten metre walk at a self-selected speed. Two orthopaedic surgeons evaluated the limping of each video in two different grading sessions with a one week delay. To avoid recall bias, the patient’s number identity was randomized and different for each grading session and each rater. The weighted Cohen’s Kappa coefficient was used to quantify the intra- and inter-reliability. The reliability of three components was studied: the presence of limping, its severity, and the compensation type.

**Results:**

For all components, the agreement for intra-rater reliability ranged from moderate to strong and from none to moderate for the inter-rater reliability.

**Conclusion:**

These results do not encourage the use of HHS-limping sub-score for data involving different raters in both clinical and research contexts. It calls for improved consensus on limping definitions or the creation of objective measures.

**Supplementary Information:**

The online version contains supplementary material available at 10.1007/s00264-023-06082-4.

## Introduction

Limping is a gait disorder described as an abnormal gait pattern that is frequently reported in patients undergoing total hip arthroplasty (THA) [1, 2]. Pain is one of the main causes of limping in these patients and is responsible for compensatory mechanisms such as the Duchenne limp pattern [3]. This pattern is characterized by an exaggerated lateral bending of the trunk towards the affected limb during gait, to reduce the pain. Pauwel’s balance can explain this compensation: the centre of mass of the trunk is shifted above the hip joint centre [3], this reduces the force required by the abductor muscles to stabilize the pelvis, which in turn lowers the mechanical burden of the hip joint and results in pain relief [4]. Abductor weakness is also a common cause of limping [5]. As a consequence of abductor weakness, THA patients can present a pelvic drop which characterizes the Trendelenburg limping pattern. It is interesting to note that the pelvic drop can be compensated by the Duchenne limping pattern. Duchenne limping pattern can therefore be the result of both pain and abductor weakness in severe cases of Trendelenburg limping. The limping and various compensations result in increased energy expenditure [6] and an accelerated process of wear and tear at the hip joint [7]. With higher functional limitations, limping was also reported to reduce the postoperative quality of life [8] and postoperative patient satisfaction [2].

Although limping is an important symptom to characterize the gait pattern of patients with hip osteoarthritis or THA, it is rarely reported in the studies. This may be because there is no specific questionnaire or scale for its assessment. Most of the time, the evaluation of limping is included in the general function and pain assessment of the hip. The Harris Hip Score (HHS) is widely used in patients with hip disorders and is validated and reliable to quantify pain, function, deformity, and range of motion [9]. The limping is included in the function-gait domain of the HHS and is graded with a Likert scale between 0 and 3 (0= none; 1=lightly; 2=moderate; 3=severe). Several studies used this sub-score (or similar scales) independently to investigate the influence of limping severity on different outcomes such as patient satisfaction or the effectiveness of the surgery [1, 2, 8]. However, although the global HHS score is deemed reliable, the reliability of the limping sub-score has not been specifically investigated. Therefore, assessing the reliability of this sub-score is necessary to fully understand the results of past and future studies focusing on limping and to gain confidence in this commonly used clinical tool.

This study aimed to evaluate the intra- and inter-rater reliability within-day of the Harris Hip limping sub-score. Three components were investigated: (1) the reliability of limping status independently of the severity (limping/no limping), (2) the reliability of the limping severity, and (3) the reliability of the type of limping compensation (Duchenne/Trendelenburg).

## Materials and methods

This retrospective cohort study was performed in line with the principles of the Declaration of Helsinki. Approval was granted by the Cantonal Research Ethics Committee (Geneva, Switzerland) on October 26th (CCER: 2017-00817).

### Participants

Thirty patients undergoing THA were randomly selected retrospectively from a research project (starting in 2017) of the Geneva University Hospitals including a clinical gait analysis. As inclusion criteria, patients were included if they were between 30 and 80 years old, programmed for a primary elective THA (e.g. due to osteoarthritis), with an anterior, lateral, or posterior surgical approach, and with a ceramic-on-polyethylene implant device.

Thirty asymptomatic participants (Controls) were also included in the study to increase the number of participants without limping. They were included if they were between 30 and 80 years old and had no osteoarthritis or previous THA. Exclusion criteria for both groups were the following: Incapability to walk ten m without assistance, previous arthroplasty of the lower limbs, resident in a retirement home/special care institution, and neurological, muscular, or orthopaedic problems (other than THA-related) that could affect gait parameters.

### Limping evaluation

Limping was assessed on an ordinal scale of 0 to 3 (0 = none; 1 = slight; 2 = moderate; 3 = severe) according to the HHS limping sub-score. Additionally, a qualitative evaluation of limping was performed by reporting the presence of Trendelenburg limping pattern, Duchenne limping pattern, or “other” if the limping did not fall within the two previous categories. The Trendelenburg limping pattern was defined as “a contralateral pelvic drop during a single leg stance [10], and the Duchenne limping pattern was defined by trunk lean towards the affected stance limb with the pelvis stable or elevated on the swinging limb side during unipodal phase [11]. Note that this qualitative evaluation is not included in the HHS.

### Design of the study

**Fig. 1:**
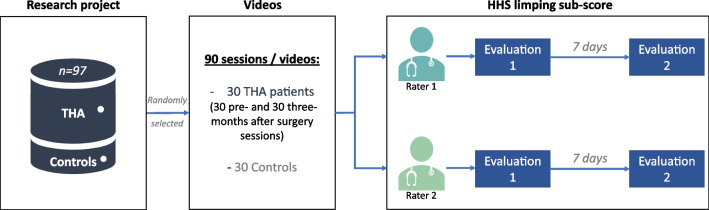
Flowchart of the study. THA: total hip arthroplasty; HHS: Harris Hip Score

The patients performed a clinical gait analysis before and three months after surgery (2 sessions of analysis) and the control group only once. This analysis consisted of 2D video recordings and 3D motion analysis of the full body during gait and various functional tasks (including sit-to-stand, timed up-and-go, and unipodal balance). This led to a database of 90 videos (front and back view side-by-side) of participants walking at a self-selected speed. The face of the participants was blurred and the patient’s identity number and affected side were reported on the videos (example: “Subject n°1 - LEFT”) as shown in the supplementary data 1. A random side was affected to control participants to blind raters from the presence of a control group (see Fig. 1).

### Raters

Two senior orthopaedic surgeons (MG, AD) performed the limping evaluation on each video in two different sessions with a one week delay. They had more than seven years of experience and performed approximately 75 THA per year. In the clinical routine, they performed an examination including a limping evaluation with the HHS-limping sub-score on patients pre- and post-surgery.

### Statistical analysis

Three components were assessed for reliability as follows: (1) the limping status, (2) the limping severity, and (3) the type of limping compensation. The reliability of the limping status (component 1) was evaluated on all sessions of the THA patients and Controls (90 videos) considering the status as limp for scores superior or equal to 1 and as no limp for scores equal to 0. From a clinical point of view, we consider more relevant to highlight the *moderate & severe* limping severity. Thus, a second limping status was set considering the status as limp for scores superior or equal to 2 and as no limp for scores inferior or equal to 1. The reliability of the limping severity (component 2) was evaluated only on the THA patients (60 videos: 1 video per session). The type of limping compensation (component 3) was evaluated only on videos in which a score superior or equal to 1 was reported by each rater in each session (25 videos included and 35 videos excluded).

The weighted Kappa coefficient was used to quantify the intra- and inter-reliability [12]. The interpretation of the Kappa coefficient was classified as follows: 0–0.20 as none, 0.21–0.39 as minimal, 0.40–0.59 as weak, 0.60–0.79 as moderate, 0.80–0.90 as strong, and above 0.90 as almost perfect agreement [12]. Confidence intervals were also reported. The percentage of agreement can be also used to quantify the reliability but it does not take into account the possibility that the raters guess the score, while the Kappa coefficient does [12]. It is nevertheless reported in the supplementary Data 2.

## Results

**Fig. 2:**
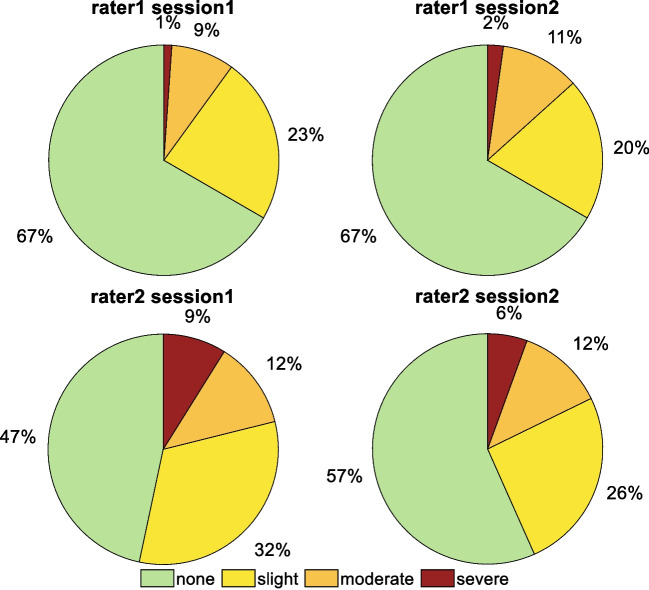
Proportion of limping severity for each rater and session of evaluation

Characteristics of the participants are presented in Table 1. Because the anterior approach is by far the most commonly used approach in the institution, only patients with anterior approach happened to be randomly included in the study. Among the four evaluations, limping was observed in 41% of the videos (7% of the Controls; 71% and 47% of the patients for pre- and post-3-month sessions, respectively). Note that limping in Controls was observed only for one rater either slight (*n*=7) or moderate (*n*=1). The proportion of each rater for each session of evaluation is presented in Fig. 2.

**Table 1:** Characteristics of the participants

	THA (*n*=30)	Controls (*n*=30)
Female (n, %)	12 (40%)	16 (53%)
Age (*years)*	63.2 ± 11.5	67.1 ± 7.6
Height (*m)*	1.66 ± 0.86	1.65 ± 0.10
Weight (*kg)*	78.5 ± 17.0	67.8 ± 11.3
BMI (*kg/m*^*2*^*)*	28.1 ± 4.0	24.8 ± 4.2
Surgery approach	Anterior (*n*=30)	-
Implant type (n, Cup-Stem)	‐ 6, Pinnacle^®^-Actis^™^ (DePuy Synthes) ‐ 4, Pinnacle^®^-Corail^®^ (DePuy Synthes) ‐ 19, VersafitCup^®^ – Quadra^®^ (Medacta) ‐ 1, PolarCup^™^ (Smith&Nephew)-Corail^®^ (DePuy Synthes)	-

*Data are presented as n(proportion) for gender and mean ± standard deviation for the other parameters*

**Fig. 3:**
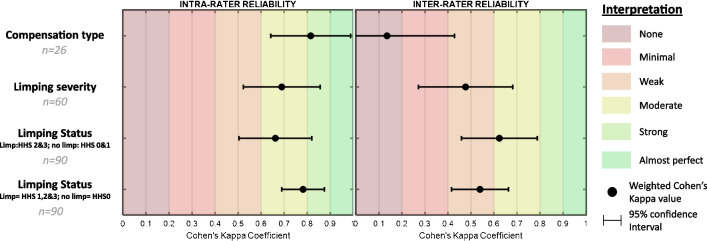
Weighted Cohen’s Kappa value for Intra- and inter-rater reliability of all limping components. HHS: Harris Hip Score limping sub-score; n: number of sessions included; interpretation: interpretation of the reliability

Concerning the limping status 1 (*none vs. slight, moderate & severe*) (component 1), Cohen’s Kappa was 0.782 [0.689 to 0.875] and 0.539 [0.415 to 0.663] for intra- and inter-rater reliability, respectively (Fig. 3). For limping status 2 (*none & slight vs. moderate & severe*) Cohen’s Kappa was 0.662 [0.503 to 0.820] and 0.624 [0.459 to 0.789] for intra- and inter-rater reliability, respectively (Fig. 3).

Concerning the limping severity (component 2), only THA patients were included in the analysis (i.e. 60 sessions/videos). Cohen’s Kappa was 0.726 [0.587 to 0.866] and 0.534 [0.359 to 0.709] for intra- and inter-rater reliability, respectively (Fig. 3).

Concerning the limping compensation type (component 3), 25 sessions from 18 different THA patients were analyzed. Cohen’s Kappa was 0.846 [0.639 to 1.000] and 0.137 [0.000 to 0.463] for intra- and inter-rater reliability, respectively (Fig. 3).

The confusion matrix and detailed results of each analysis are presented in the Supplementary Data 2.

## Discussion

This study investigated the intra- and inter-rater reliability of the HHS-limping sub-score. The reliability of three components was evaluated: limping status, limping severity, and compensation type. Results showed intra-rater reliability ranging from moderate to strong (0.77< k < 0.89) and inter-rater reliability ranging from none to moderate (0 < k < 0.62).

For all components, the intra-rater was greater than the inter-rater reliability. This result is commonly reported in the literature because the between-rater variability is eliminated in intra-rater reliability [13]. The intra-rater reliability result is close to the reliability of the HHS (global score) [9]. Indeed, the HHS showed excellent intra-rater reliability, especially for the domain of function (r>0.93), which includes the limping sub-score [14]. The difference could be related to the reliability calculation methods (correlation vs. Cohen’s Kappa coefficient). However, the HHS showed a strong to almost perfect inter-rater reliability (Cohen’s Kappa Coefficient of 0.82 to 0.91) [15], higher than the HHS-limping sub-score. The global score is composed of ten items for a total score of 100. Limping is one of these and represents only 11% of the total score [9]. The reliability of the other items, especially the pain (44% of the score) may compensate for the weak reliability of the HHS-limping sub-score.

The quantitative evaluations of the limping showed moderate to strong intra-rater agreement while inter-rater agreement was weak to moderate. In other words, the assessment of limping status and its severity level using the HHS-limping sub-score is adequate when the evaluation is performed by only one clinician, but less suitable when the assessment is performed by multiple clinicians.

It is also interesting to note that distinguishing *moderate & severe* limping from *none & slight* limping presents better reliability than *none* from *slight, moderate & severe* limping. For limper vs. non-limper group analysis, we suggest using the *moderate & severe* scores as limper and *none & slight* scores as non-limper when different raters are involved. This reinforces the need to clearly describe the origin of the data and the categories of limping that are used/combined in clinical follow-up and research studies using data from a registry or database involving different clinicians.

Concerning the qualitative evaluation of limping (not included in the HHS), results showed strong intra-rater reliability but the inter-rater agreement was also qualified as none to weak. This result is partially consistent with the literature. Indeed, the Trendelenburg test agreement was reported to be none to weak in patients with hip pain [16]. It suggests that the interpretation of the Trendelenburg limping pattern and Duchenne limping pattern varies between clinicians. This could indicate a need to improve the consensus on the definition of limping signs in THA patients. As an example, during videotaped observation of gait, Dürregger et al. (2020) reported an intra-class correlation for inter-rater reliability ranging from 0.47 to 0.88 for a positive Trendelenburg’s sign set over 8° and from 0.76 to 0.92 for a positive Duchenne’s compensation set over 10° [17]. In the present study, no specific threshold was given to the rater which can explain the low inter-rater reliability. Another solution could be found in the development of tools for an objective assessment of limping based on motion capture and biomechanical outcomes which would reduce the between-rater variability. However, considering clinical constraints (time, cost, workload...), this assessment must be fast, inexpensive, and easy to use. Using the Instrumented Time Up and Go (also named iTUG) test with inertial measurement units could be an interesting pathway for clinical use [18].

### Limitations

This study presents several limitations. The limping assessment was performed on videos that could be different than seeing the patient in real life but, video analysis offers the possibility to play the video several times or frame by frame, which can improve the evaluation. Moreover, as previously reported, the percentage of agreement and the Cohen’s Kappa coefficient could individually lead to different conclusions on inter-rater reliability. It was suggested that if raters are well trained and have few risks of guessing the score, the percentage of agreement can be used. Despite the surgeons of the present study being experienced, the HHS does not include a clear definition of the severity level (slight vs. moderate vs. severe) which can lead to a guessing score and different interpretations between clinicians. Nevertheless, similar results using the percentage of agreement (Supplementary material 2) on limping severity were found. Regarding the limping type, the frontal viewpoint of the video only allowed the raters to estimate deviations in the frontal plane. Different types of limping characterized by deviations in other planes, e.g. hip extension deficits for the sagittal plane, may have been missed and not reported in the “other” category of limping [19].This may therefore have influenced the reliability of the type of limping.

## Conclusion

This study found a moderate to strong intra-rater agreement and a none to moderate inter-rater agreement for the HHS-limping sub-score. These results highlight the limitations of using the HHS-limping sub-score for data involving different raters in both clinical and research contexts. For a limper vs. non-limper group analysis, we suggest using the *moderate & severe* scores as limper and *none & slight* scores as no limper when different raters are involved. This study suggests clarification of the definition of limping (presence/absence and severity level) and the training of the raters according to the same definitions. Another solution could be the development of an objective outcome measure based on biomechanical parameters which would limit the influence of the between-rater variability.

### Supplementary Information

Below is the link to the electronic supplementary material.

Supplementary file1 (MP4 13128 KB)

Supplementary file2 (PPTX 471 KB)
