# Supplementary material for: Reliability of the Harris Hip limping sub-score in patients undergoing total hip arthroplasty
Source: Int Orthop. 2024 Jan 13;48(4):991–6. doi: 10.1007/s00264-023-06082-4 (PMC10933184; doi:10.1007/s00264-023-06082-4)
Supplement: Supplementary file 2 — Supplementary file2 (PPTX 471 KB) [file 264_2023_6082_MOESM2_ESM.pptx]

## Slide 1
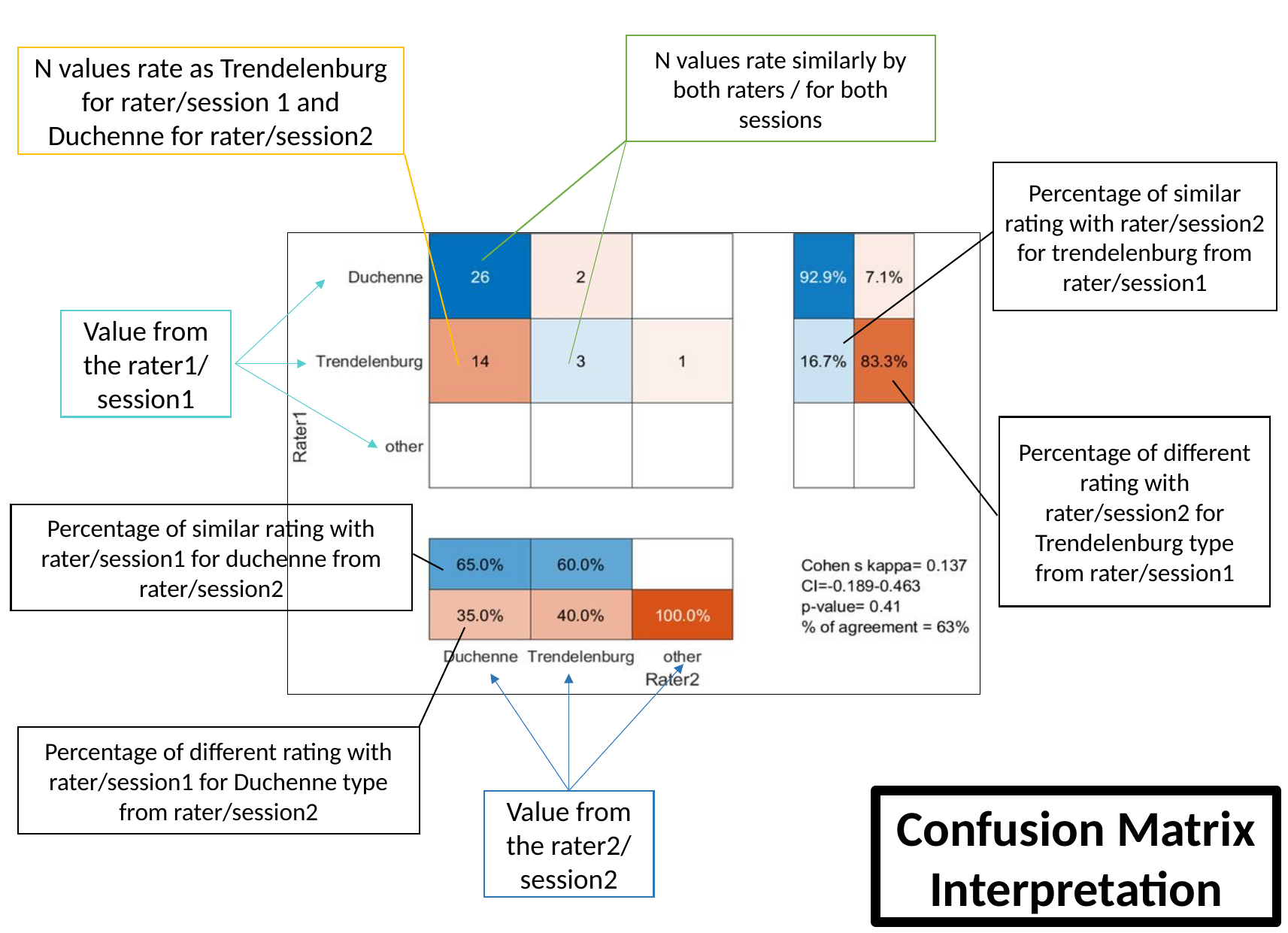

N values rate similarly by both raters / for both sessions
N values rate as Trendelenburg for rater/session 1 and Duchenne for rater/session2
Percentage of similar rating with rater/session2 for trendelenburg from rater/session1
Value from the rater1/ session1
Percentage of different rating with rater/session2 for Trendelenburg type from rater/session1
Percentage of similar rating with rater/session1 for duchenne from rater/session2
Percentage of different rating with rater/session1 for Duchenne type from rater/session2
Value from the rater2/ session2
Confusion Matrix Interpretation

## Slide 2
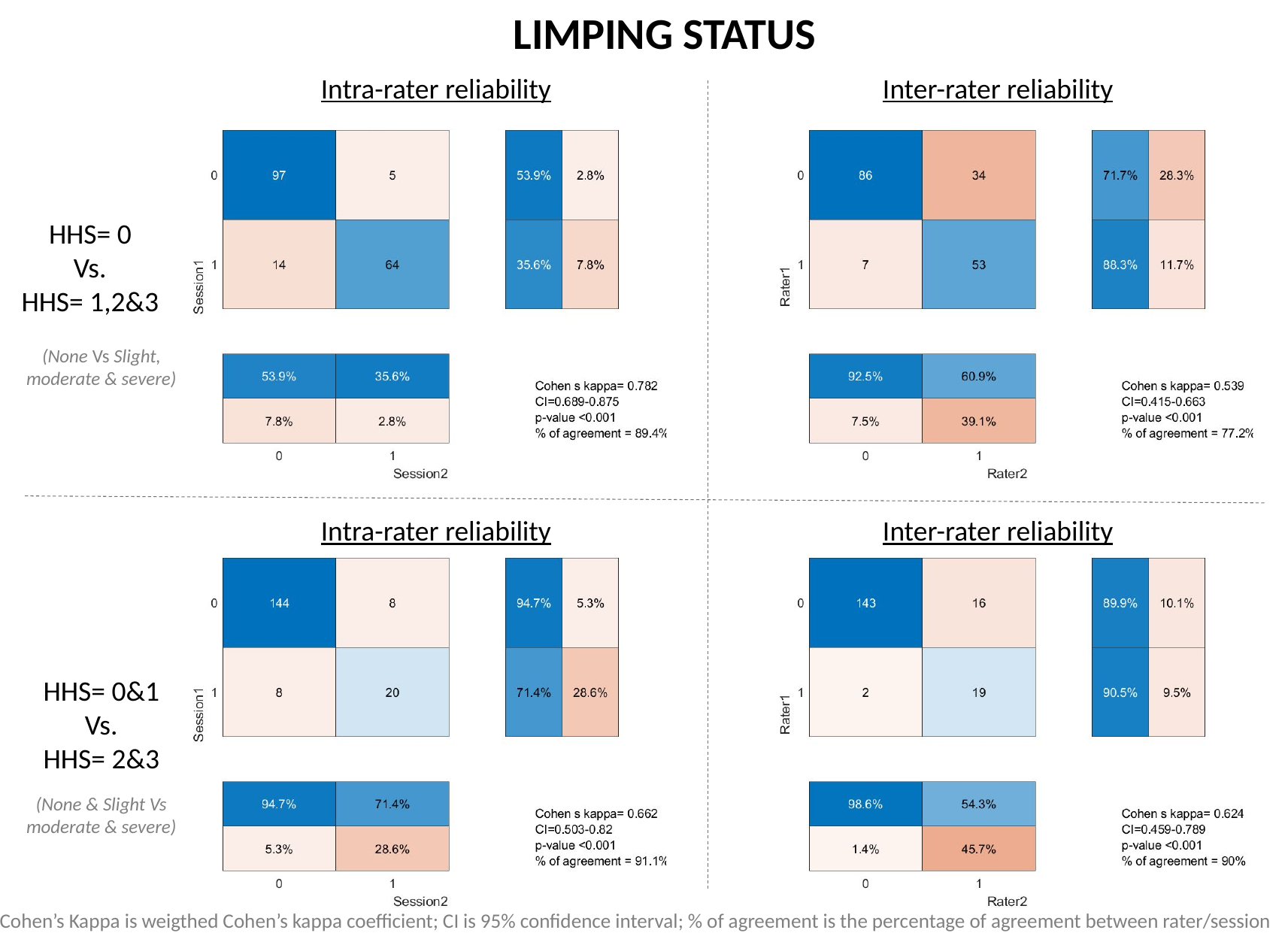

LIMPING STATUS
Intra-rater reliability
Inter-rater reliability
HHS= 0
Vs.
HHS= 1,2&3
(None Vs Slight, moderate & severe)
Intra-rater reliability
Inter-rater reliability
HHS= 0&1
Vs.
HHS= 2&3
(None & Slight Vs moderate & severe)
Cohen’s Kappa is weigthed Cohen’s kappa coefficient; CI is 95% confidence interval; % of agreement is the percentage of agreement between rater/session

## Slide 3
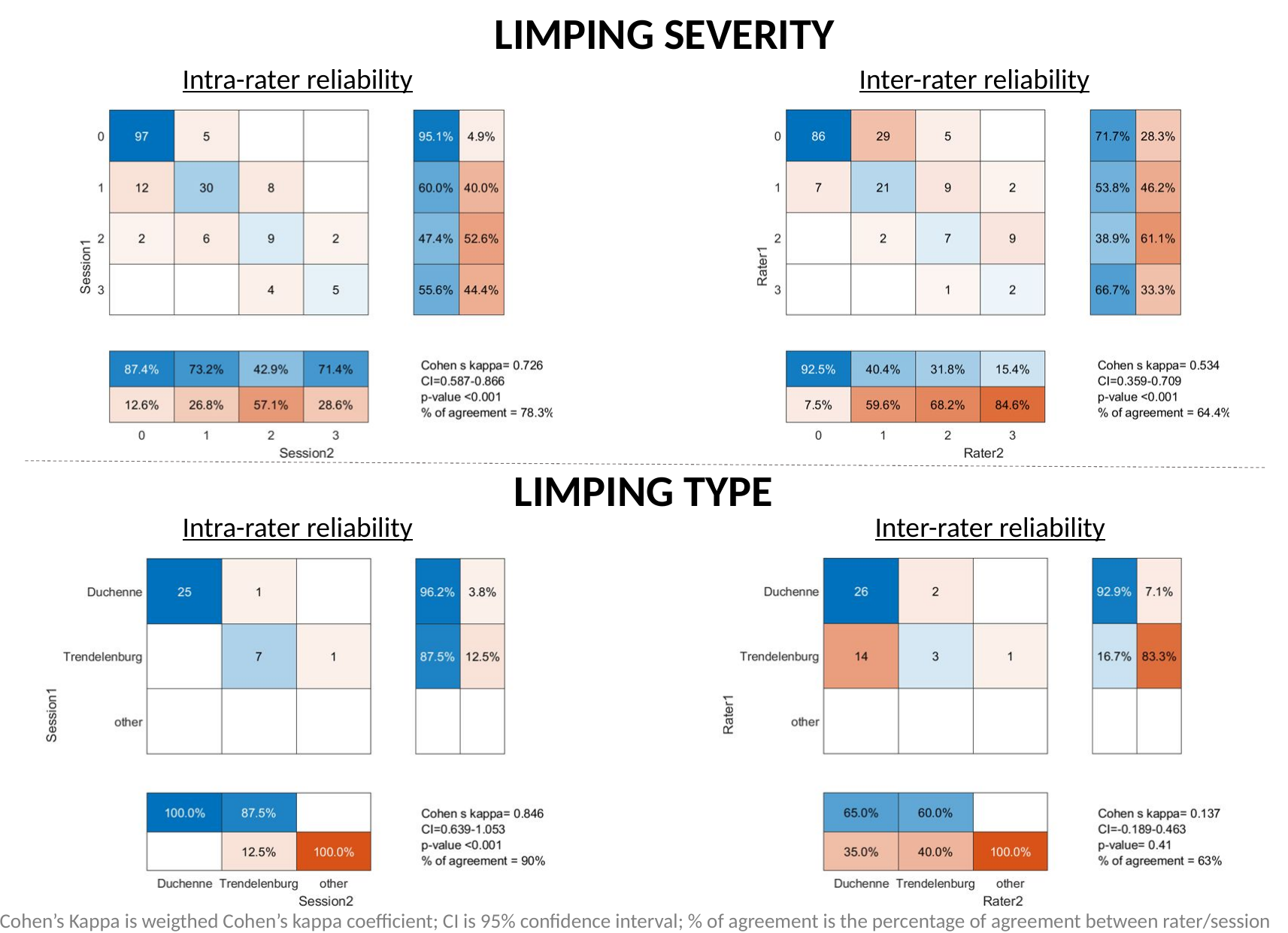

LIMPING SEVERITY
Intra-rater reliability
Inter-rater reliability
LIMPING TYPE
Intra-rater reliability
Inter-rater reliability
Cohen’s Kappa is weigthed Cohen’s kappa coefficient; CI is 95% confidence interval; % of agreement is the percentage of agreement between rater/session
